# The Phanerozoic climate

**DOI:** 10.1111/nyas.14920

**Published:** 2022-11-03

**Authors:** Nir J. Shaviv, Henrik Svensmark, Ján Veizer

**Affiliations:** ^1^ Racah Institute of Physics Hebrew University of Jerusalem Jerusalem Israel; ^2^ National Space Institute Technical University of Denmark Lyngby Denmark; ^3^ Department of Earth and Environmental Sciences University of Ottawa Ottawa Ontario Canada

**Keywords:** atmospheric ionization, paleoclimate, Phanerozoic, seawater geochemistry

## Abstract

We review the long‐term climate variations during the last 540 million years (Phanerozoic Eon). We begin with a short summary of the relevant geological and geochemical datasets available for the reconstruction of long‐term climate variations. We then explore the main drivers of climate that appear to explain a large fraction of these climatic oscillations. The first is the long‐term trend in atmospheric CO_2_ due to geological processes, while the second is the atmospheric ionization due to the changing galactic environment. Other drivers, such as albedo and geographic effects, are of secondary importance. In this review, we pay particular attention to problems that may affect the measurements of temperature obtained from oxygen isotopes, such as the long‐term changes in the concentration of δ^18^O seawater.

## Graphical abstract

We review the long‐term climate variations during the last 540 million years (Phanerozoic Eon). We first summarize the geological and geochemical datasets available for reconstruction of long‐term climate variations. We then explore the main drivers of climate that appear to explain a large fraction of these climatic oscillations. The first is the long‐term trend in atmospheric CO_2_ due to geological processes, while the second is the atmospheric ionization due to the changing galactic environment.

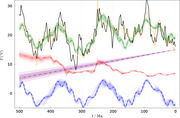

## INTRODUCTION

Global climate change, on all time scales, is a fundamental aspect of Earth evolution. These climate variations have been caused by a range of drivers, which can be either intrinsic or extrinsic to the Earth's system. For example, on very long time scales, the solar output has slowly increased,^1^, ^2^, ^3^, ^4^ giving rise to the so‐called young faint sun paradox.^5^, ^6^, ^7^, ^8^, ^9^ Simply stated, “If the Earth received less energy from the Sun in the early Precambrian, then why was the Earth so warm back then?” One suggested answer is that during the Precambrian the atmospheric composition gradually changed from a strongly reducing oxygen‐deficient atmosphere with large amounts of greenhouse gases (such as CO_2_, CH_4_, and NH_3_) to an oxygen‐rich atmosphere with significantly lower concentrations of greenhouse gases. The greater concentration of greenhouse gases kept the Earth warm during the early Precambrian. Subsequently, the gradual increase in solar radiation was balanced by decreasing amounts of greenhouse gases.^9^, ^10^, ^11^

On much shorter time scales, we still find that global climate change is governed by intrinsic and extrinsic factors. For example, the eruption of Large Igneous Provinces (LIPs) can add massive amounts of greenhouse gases to the atmosphere that warm the Earth,^12^ whereas bolide impacts can generally cool the Earth on both short^13^, ^14^ and intermediate time scales.^15^

Over very short time scales (20–100 Ka), changes in Earth's orbital parameters, known as the Milankovitch cycles, affect the amount of insolation reaching high latitudes.^16^, ^17^, ^18^, ^19^ This in turn affects the growth or waning of ice sheets, which modulates the terrestrial energy budget by changing the Earth's albedo, thus impacting ocean temperature and global levels of atmospheric CO_2_.^20^, ^21^, ^22^, ^23^ Note, however, that Milankovitch's theory does have its caveats; the main one is related to the dominance of the 100 Ka cycle. Consequently, alternative explanations have also been suggested, such as the changes in Earth's orbital inclination.^24^

Here, we shall concentrate on the intermediate time scale of 100's of millions of years and review climate change during the Phanerozoic Eon.^25^, ^26^, ^27^, ^28^, ^29^, ^30^, ^31^, ^32^, ^33^ This is the time interval when complex life arose, producing numerous fossils that can be analyzed by chemical methods to describe global changes. The Phanerozoic is also characterized by a relatively stable atmospheric composition. We will review global climate change over this time scale, and show that the observed climatic variability is governed by a combination of both intrinsic and extrinsic drivers.

In this review, we will not consider variations shorter than a few million years. As mentioned above, these are governed by orbital forcing, or by other short‐term causes, such as large‐scale volcanic eruptions or the out‐gassing of methane from the deep sea as postulated, for example, for the Palaeocene‐Eocene Thermal Maximum.^34^ We will also avoid discussion of regional drivers (e.g., climate change caused by the formation of mountain ranges, even if they may have a global effect) and will restrict our discussion to climate drivers on a global scale.

In the second part, we will review other models that describe how the global average temperature has changed during the Phanerozoic. These models are based on both geochemical and nongeochemical evidence, and we will point out the advantages and problems with each method. We will then combine these estimates of paleotemperature to produce a more reliable Phanerozoic temperature curve that we will then explain in terms of the radiative drivers.

In the third part, we will review the principal intrinsic and extrinsic radiative drivers and explain how they affect the global temperature. The former, primarily greenhouse gases, vary due to geological activity (i.e., volcanic eruptions and chemical weathering). The latter include long‐term variations in the solar output as well as changes in the galactic cosmic ray (CR) flux.

We will then continue in the fourth part to review the chronology of these climate drivers and in the fifth part to compare the predicted temperatures to the Phanerozoic paleotemperatures. We will demonstrate that the aforementioned intrinsic and extrinsic drivers can explain a significant portion of the observed temperature variations. Moreover, this comparison enables us to settle the protracted debate regarding the interpretation of ancient oxygen isotopic measurements of temperature. There is clearly a long‐term, secular drift in oxygen isotope values during the Phanerozoic that extends to the Archean, 3 billion years ago.^35^ Some authors claim that this trend is a result of postdepositional (diagenetic) recrystallization of samples that becomes more severe with their age.^36^, ^37^ Others argue that the trend reflects the evolving oxygen isotopic composition of seawater.^28^, ^30^ This trend must be removed (i.e., detrended) to obtain realistic temperature measurements. This is elaborated on in the second section of the second part.

This review is by no means comprehensive. Readers are directed to additional reviews that have recently appeared on the topic of Phanerozoic paleotemperatures. Scotese^31^ reviews Phanerozoic climate with an emphasis on lithologic indicators of climate. The second type of studies^29^, ^32^ reviews the temperature record derived from oxygen isotope measurements obtained mostly from phosphatic, rather than carbonate, shells. The review by Goddéris et al.^33^ combines observational data with climate models for the different Phanerozoic epochs. These reviews are all complementary to the discussion presented here. Our essay places an emphasis on modeling Phanerozoic climate as a whole, pinpointing the role of the main climate drivers, both intrinsic and extrinsic to the Earth's system, that operated continuously throughout planetary history.

## RECONSTRUCTING THE PHANEROZOIC CLIMATE

Generally speaking, there are two types of temperature and climate reconstructions over geological time scales. One type, based on lithological indicators of climate, such as coals, evaporites, bauxites, and tillites,^35^, ^38^ aims to reconstruct the Earth's past climatic zones (Köppen belts) and the pole‐to‐equator temperature gradient.^31^ The second reconstruction is geochemical and uses oxygen isotope measurements of paleotemperature.^26^, ^29^, ^30^, ^36^, ^37^ Both methodologies have important advantages and disadvantages.

### Qualitative lithological proxies

Lithologic indicators of climate can be used to map the ancient climatic zones (Köppen belts). From the equator to the poles, the major Köppen belts are: (1) tropical rainforests; (2) desert belts; (3) warm temperate grasslands and forests; (4) seasonally warm/cold temperate regions; and (5) frigid polar regions. By mapping the ancient extent of these Köppen belts, it is possible to estimate global average temperatures.^31^

Compared to the isotopic climate records described below, the downside of the lithological reconstruction of paleotemperature is that it is very hard to achieve high temporal resolution. The paleogeographic maps of Scotese and collaborators^31^ cover ∼100 slices of the Phanerozoic, with an average duration of ∼5 million years. On the other hand, it has one very clear and significant advantage. It does not suffer from any long‐term secular biases that may affect the δ^18^O database, nor is it indirectly affected by any of the drivers themselves.

### Quantitative geochemical proxies

The shells of a variety of organisms (brachiopods, foraminifera, mussels, and conodonts) that live in diverse ecological environments are made of calcium carbonate, calcium phosphate, and siliceous minerals. The ratio between the ^18^O to ^16^O isotopes found in these shells depends on multiple environmental factors. The primary factor is the temperature of the ambient water.^39^, ^40^ The relationship between δ^18^O and temperature is such that a compositional change in δ^18^O of −1‰ corresponds to about a +4°C increase in the temperature at the time of precipitation. However, the relation also depends on the salinity and the ratio of ^18^O to ^16^O in seawater. Salinity depends on paleoclimate (arid vs. humid climates), and the ratio of ^18^O to ^16^O in seawater depends in part on the amount of water locked in the continental ice caps. For example, assuming that during glacial maximums, the amount of water locked in continental ice is about twice the volume of the Antarctic ice cap (∼30 million km^3^) and that no polar ice caps existed during warm intervals, the expected variations from the waxing and waning of ice caps would be about 2‰.^41^

While the δ^18^O reconstruction has a clear advantage in providing quantitative, high‐resolution data (with appropriate environmental corrections), there are three major caveats that must be taken into account.

**FIGURE 1:**
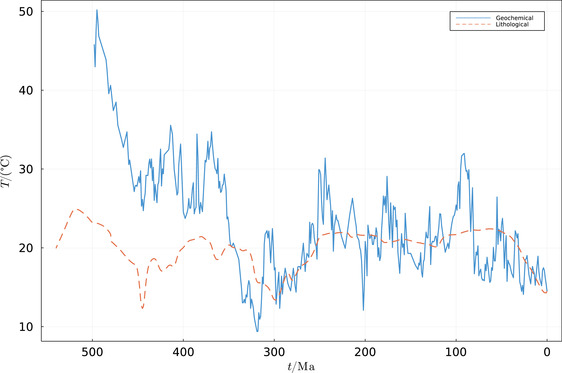
The underlying reconstructions used in the present analysis. The geochemical reconstruction^29^ is depicted with a solid line, while the lithological reconstruction^44^ is depicted with a dashed line. Note that whereas some of the gross features are similar, a notable difference is the extreme geochemically based reconstructed temperatures derived for the early Phanerozoic.

**FIGURE 2:**
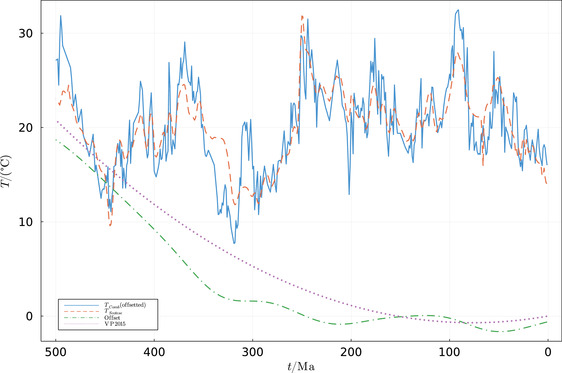
The Scotese et al.^44^ reconstruction (dashed) is based on lithological data on long‐time scales and oxygen isotope data for medium‐time scales (10–20 Ma). The lithological/geochemical combined reconstruction we use here (i.e., Equation 1) is the solid line. The difference between the nondetrended isotopic reconstruction of Song et al.^29^ and our combined reconstruction is plotted with the dash‐dotted line. It depicts the systematic secular offset in isotope data discussed above. Without this correction, the data taken at face value would require unrealistically hot temperatures for the ancient oceans.^29^ Note that the systematic correction of −4°C per 1‰ δ^18^O suggested by Veizer and Prokoph^28^ for the oxygen isotope record of carbonate shells (dotted) is practically identical to the one employed here and based mostly on phosphatic shells.

Quite early on, it was realized that there is a 4–8‰ decline in δ^18^O for progressively older Phanerozoic measurements,^42^, ^43^ and double that for the early Precambrian.^35^ If taken at face value, it would imply unrealistically high ocean temperatures of 30°C during the extensive Ordovician/Silurian glaciations, and 50°C during the late Cambrian (e.g., see Ref. 29 and Figure 1), and almost near‐boiling oceans during the Precambrian, even at times of massive glaciations.^45^ The general wisdom for the last four decades, therefore, implied that a diagenetic process superimposed itself over temperature fractionation,^36^, ^37^ making the data useless for long‐time scale temperature reconstruction. This changed with Veizer et al.,^26^ who compiled thousands of oxygen isotope measurements of well‐preserved low‐Mg calcitic fossils over the entire Phanerozoic. They documented a clear cyclic pattern of oscillations superimposed on a secular trend that was consistent with climate variations deduced from lithological indicators, demonstrating that the original temperature imprint remained in the fossils. It should be noted that the same samples yielded also additional measurements that were entirely consistent with independent studies of other laboratories, such as isotopes of carbon and radiogenic strontium,^26^ stable strontium,^46^ sulfur,^47^ calcium,^48^ and Sr/Ca elemental ratios.^49^ It is difficult to argue that oxygen, the dominant element in the calcite structural cell, was completely diagenetically replaced, while all other major and minor elements and isotopes remained untouched. Moreover, the long‐term secular trend was documented not only for carbonates, but also for siliceous and phosphatic samples. These isotopic changes cannot be, therefore, explained in terms of diagenesis because these three mineral phases differ in their stability and isotopic fractionation factors. Their time series records, such as the ones discussed later in Figure 2, would then show divergent trends in response to variable sensitivity to resetting processes. The long‐term secular trend is more likely primary, reflecting a nonmonotonic, slow (on the order of 0.01‰/Ma) oxygen isotopic evolution of seawater, possibly due to the slowing of the plate tectonics that buffers the oceanic δ^18^O.^30^

The second caveat is more delicate. Zeebe^50^ pointed out that ocean acidity can produce an offset in the δ^18^O data that mimics temperature variation: *∆T_pH_ = bs∆pH* with *b* being the ratio between oxygen fractionation and temperature, that is, *b* ∼ 4°C per ‰, and *s* being the ratio between δ^18^O and pH variations, which from theory and experiments appears to be around *s* = −1.42‰ per unit pH.^51^ Royer et al.^52^ then suggested that the Phanerozoic temperature reconstruction would be affected by the atmospheric concentration of CO_2_, which modifies oceanic acidity. The implication was that part of the temperature response to CO_2_ warming is countered by corresponding pH variations. In fact, for their canonical values,^52^ it was shown that for climate sensitivity of ∼1.5°C increase per CO_2_ doubling, there should actually be no correlation between the δ^18^O‐derived temperature and the CO_2_.^53^ This could explain the lack of correlation between CO_2_ and temperature found in several cases.^27^, ^41^, ^54^ Note also that the scenario of Royer et al.^52^ involves multiple assumptions about processes that relate ocean acidity and alkalinity to pCO_2_. These assumptions should be taken with a grain of salt. In the following sections, we will try to estimate this bias empirically by comparing the lithological and isotopic reconstructions of paleotemperature. We define the “unbiased” temperature as the real temperature, while the “biased” temperature is the one modified through the pH bias.

The third caveat relates to the fact that most fossils producing hard mineral shells lived in tropical and warm temperate habitats (<40° latitude). The Phanerozoic oxygen isotope temperature record, therefore, reflects the temperature of low‐latitude seas rather than the entire global ocean (see Ref. 31 for a detailed discussion of the issue and scaling).

### A combined temperature reconstruction

Given that the caveats of the different types of data sets are entirely independent, one could, in principle, combine lithological and geochemical data into a new temperature reconstruction that avoids the aforementioned problems. This was realized by Scotese et al.,^44^ who generated a Phanerozoic temperature reconstruction that is based on both lithological indicators of climate on long‐time scales and oxygen isotope data on the intermediate 10–20 Ma time scales. Interestingly, Scotese et al.^44^ compared their Phanerozoic temperature model to short‐term LIP eruptions and bolides impacts, finding that 19 of 21 LIP eruptions broadly match the times of elevated temperatures, while 18 of 22 of the major bolide impacts correspond to the times of global cooling. In this essay, we combine the same lithological and geochemical data sets (Figure 1) using a different technique, described below, while considering that some of the proxy data can be systematically biased. We obtain a similar result (Figure 2).

If we define a smoothing kernel:

(1) $$K\left(t,t^{\prime }\right)=\frac{1}{\sqrt{2\pi }\sigma_{t}}\exp\left(-\frac{{\left(t-t^{\prime }\right)}^{2}}{2\sigma_{t}^{2}}\right),$$

with *σ_t_* taken to be 30 million years, we can combine the lithological reconstruction^31^ *T_L_* with isotopic reconstruction^29^ *T_I_* (Figure 1) as follows:

$$T_{C}=\underset{-\infty }{\int^{\infty }}K\left(t,t^{\prime }\right)T_{L}dt^{\prime }+\left(T_{I}-\underset{-\infty }{\int^{\infty }}K\left(t,t^{\prime }\right)T_{I}dt^{\prime }\right).$$

This definition recovers *T_I_* for *σ_t_* → ∞ and *T_L_* for *σ_t_* → 0. In order to avoid edge effects, we carry out the integral from 0 to *t_max_*, normalize *K* appropriately, and correct for the asymmetric boundaries by fitting a linear slope and correcting the expected bias in the integral.

Figure 2 compares our combined temperature reconstruction (this study, solid line) with the Scotese et al.’s combined model^31^ (dashed line). Both curves use the same input data *T_I_* and *T_C_*. The differences between the two curves are due to the fact that Scotese et al.^31^ (1) used a σ of 55 million years, which reduced the overall amplitude of the curve; (2) employed the Savitsky–Golay fitting technique to smooth the curve; and (3) modified the curve to better agree with geological and paleontological constraints (most notably at 30, 66, 310, and 340 Ma). The difference between the original nondetrended isotope data of Song et al.^29^ and our combined reconstruction that effectively detrends the isotopic data is plotted as the dash‐dotted curve in Figure 2.

Our base temperature reconstruction (“this study”) will serve as the data we will analyze below. It can also be found in Table S1 and at http://www.phys.huji.ac.il/~shaviv/the‐phanerozoic‐climate.

## MAIN CLIMATE DRIVERS OF THE PHANEROZOIC

The climate drivers over the Phanerozoic can be divided into two primary groups. The first is drivers intrinsic to the Earth system, the variation of which arises due to various geological processes. The second is extrinsic drivers that depend on Earth's interaction with its celestial environment.

### Intrinsic drivers—Atmospheric composition

By far, the most important intrinsic climate driver is the changing atmospheric composition. Asymmetric diatomic molecules or any larger molecules have absorption bands in the infrared (IR) that give rise to the so‐called greenhouse effect. Increasing their atmospheric abundance increases the IR optical depth such that the surface from which the terrestrial radiation can be radiated back to space is generally higher in the atmosphere. Below this surface, a temperature gradient has to exist to advect the heat from the surface (either through convection or radiation) to the height from which the IR is emitted to space. Thus, increasing the amount of greenhouse gases implies that the temperature gradient has to exist over a larger height and the surface temperature, therefore, has to increase.

The most abundant IR‐absorbing molecule is water. However, because it is condensable it cannot be considered as a *climate driver*, since the amount in the atmosphere is the result of a climate equilibrium, not due to extrinsic processes. The next IR‐absorbing molecule in the atmosphere is CO_2_. Although some of the processes depend on the temperature (equilibrium between atmospheric CO_2_ and mostly carbonic acid in the oceans), the large variations over geological times scales depend on geological processes and not temperature (mostly volcanic activity vs. sedimentation as limestone). The effect of the greenhouse gases is usually quantified as the change in radiative forcing associated with an increase in their atmospheric abundance, in other words, how does the radiation to space change if we increase or decrease the amount of greenhouse gases but do not change the thermal profile of the atmosphere. Since the IR radiative lines are mostly saturated, the differential contribution is from the line wings such that an increase in the amount of greenhouse gases generally increases the radiative forcing only logarithmically. It is, therefore, customary to define the greenhouse effect as the change in radiative forcing associated with a doubling in the concentration of greenhouse gases. For CO_2_, the standard estimate for the radiative forcing associated with a doubling of CO_2_ is *∆F_×2_* ≈ 3.7 W/m^2^.^55^ Other greenhouse molecules that have made a contribution over geological time scales are methane and ammonia. However, beyond direct measurements in ice cores in the past million years, there is no reliable way to estimate their past atmospheric concentrations.

Other effects on the radiative budget arise from changes in the Earth's albedo. These changes include variations in the surface albedo (due to ice and vegetation) as well as due to changes in the cloud cover. One can roughly estimate the surface albedo (in particular, the contribution from ice). However, we do not consider cloud cover variations to be a climate driver (except perhaps through CRs as explained below), but instead, they are part of how the climate reacts to imposed drivers. Since we will not estimate the albedo effects, it will imply that any albedo effects that are due to a response of the terrestrial system to climate changes, such as the extent of glaciations, are going to be implicitly considered to be part of the overall climate sensitivity that we describe below.

Other climate drivers include changing geography, such as mountain ranges that affect air mass flow, or changes in oceanic circulation. It is hard, however, to assess the effects of such drivers on a global scale, let alone reconstruct them over geological time scales. We will, therefore, omit discussing them altogether. Unlike the albedo response to the climate, we do not expect these drivers to be climate‐driven. Ignoring the climatic effects of changing geography implies that some of the observed climate variations are left unexplained.

### Extrinsic drivers—Solar output and CRs

Perhaps the best‐known extrinsic driver is the Milankovitch cycles, in which the gravitational forces exerted by the sun, moon, and planets affect the orbital parameters of the Earth. Milankovitch cycles give rise to climate variations on a time scale of 20–100 Ka and, therefore, are irrelevant when considering climate change on the time scale of the Phanerozoic (10's of millions of years).

There is, however, another extrinsic driver that acts on time scales of millions of years. Although the idea has been controversial, we now know that CR flux variations have a large effect on the climate. They were first considered as the mechanism linking solar variations to terrestrial climate,^56^ but later, it was realized that they can explain climate variations over geological time scales as well.^27^, ^57^, ^58^, ^59^ With the exception of CRs with extremely high energies, most CRs are high‐energy particles that are accelerated in supernova remnants. They then diffuse throughout the Milky Way (typically over 10 Ma), eventually escaping the galaxy. Those CRs that reach the solar system are slowed down by the solar wind, but once they reach Earth's atmosphere, they generate charged particle showers that reach the lower troposphere. CRs are the dominant source of atmospheric ions. Interestingly, the flux reaching the Earth's surface decreases when solar activity is on the rise. CR production also varies according to the production rate in the solar system's vicinity.

Today, we know that this atmospheric ionization plays an important role in the nucleation of the few nanometer (nm) sized aerosols, called condensation nuclei,^60^, ^61^, ^62^ and in their growth to the ∼50 nm–sized aerosols called cloud condensation nuclei (CCNs).^63^, ^64^ These processes have been described analytically from *ab initio* physical principles; they have been observed in the lab, and they also have been seen to operate empirically *in situ*. A few examples include:

1. Forbush decreases. It is possible to decouple cosmic ray flux (CRF) variations from other changes related to solar activity (such as UV) over the time scale of days and record the physical chain of events that consequently takes place between atmospheric ionization changes and CCNs. Forbush decreases are several days–long reductions in the CRF that appear typically 1 day after large eruptions on the solar surface. The strongest Forbush decreases are associated with reductions in the number of aerosols, as well as with changes in different cloud parameters derived from cloud data sets and satellites.^65^, ^66^
2. Although the 11‐year cycle in the cloud cover could, in principle, arise from another solar link, one that is unrelated to the CR flux, the observed cloud cover changes have a CR imprint in them. The 11‐year cycle is actually a 22‐year cycle. Every 11 years, the north and south magnetic poles of the sun flip. It turns out, however, that only the CRs are sensitive to the polarity switch. This sensitivity manifests itself as an asymmetry between odd and even solar cycles—the CR minimum is flat during one cycle and becomes more pronounced during the next. Other variations, such as the UV flux or the strength of the magnetic field, are not affected by the magnetic polarity. It is notable that changes in cloud cover exhibit the same asymmetry as CRs.^67^
3. The CR/climate relationship is the only one capable of explaining the magnitude of the observed solar–climate interactions. For example, though it has been suggested that UV heating the stratosphere may be the result of increases in solar activity,^68^ global circulation models show that the net effect on the surface temperature is actually less than the variations due to the changed irradiance.^69^, ^70^ On the other hand, the apparent effect that the CRs have on cloud cover automatically explains the size of all the observed solar‐related climate variations. For example, the changes in the energy budget associated with the 11‐year cloud cover variations have the right amplitude to explain the calorimetric measurement of the solar radiative forcing.^71^, ^72^
4. Over geological time scales, there are large variations in the CR flux that have nothing to do with solar variability. Instead, they arise from the movement of the solar system through different galactic environments. A comparison between the CR flux (reconstructed over these time scales using iron meteorites) and climate (reconstructed using either sedimentation or geochemical records) demonstrates that the seven ice‐age epochs (during which Earth has had glaciations) over the past billion years have taken place when the CR flux was higher, as the theory predicts.^27^, ^57^ Over somewhat shorter time scales, one can also see 15 temporal periods when the solar system oscillates perpendicular through the galactic plane.^59^ We will consider these variations in the discussion that follows. On longer time scales, the secular decrease of the solar wind may explain part of the faint sun paradox, and long‐term star formation variations in the Milky Way may also explain why glaciations are seen only during the Phanerozoic, Neo‐Proterozoic, and the late Archean‐Huronian. These variations can be reconstructed from the age distribution of nearby stars. They may arise from tidal perturbations during perigalacticons of the Large Magellanic Cloud, and are a natural consequence of the CR/climate link as a larger star formation rate will translate to more nearby supernovae.^73^

Another very important extrinsic climate driver is the slow increase in solar luminosity. Insolation steadily increases because the average chemical weight at the solar core increases as hydrogen is fused into helium. As mentioned previously, this long‐term change in insolation has given rise to the so‐called “faint sun paradox,” that is, how could the Earth remain mostly unfrozen during most of its existence, even though the sun was much fainter in the distant past. Over the past 600 Ma, it corresponds to a 5% increase in solar radiation.^4^ It is perhaps the easiest driver to consider.

### Climate sensitivity

To the extent that we can describe the climate with a single number—the average global temperature—the climate sensitivity links this number to the change in the radiative forcing. If Earth were a perfect gray body (i.e., a black body in the IR with constant emissivity, ε, but “gray” with a finite constant albedo, *a*, in the visible), then a simple expression for the sensitivity can be derived. This is obtained by comparing the shortwave flux entering the system, *πR* ^2^ *(1 − a)S*_0_ (with *a*, *R*, and *S*_0_ being the albedo, Earth's radius, and solar constant, respectively), to the IR leaving it, *4πR* ^2^ε*σT* ^4^ (with ε, *σ*, and *T* being the IR emissivity, Stephan–Boltzmann constant, and an effective temperature describing Earth, respectively). Earth's equilibrium effective temperature can then be solved for:

$$T={\left(\frac{\left(1-a\right)S_{0}}{4\varepsilon \sigma }\right)}^{1/4}.$$

The climate system is, however, much more complicated because positive feedback changes both the albedo, *a*, and the emissivity, *ε*, in the above equation. For example, cooling the planet would increase the extent of ice cover, thus increasing the albedo and further cooling the planet. Increasing the average global temperature would increase the amount of water vapor in the atmosphere, but it would also change the cloud cover, further affecting both *a* and *ε*. Thus, it is extremely hard to calculate Earth's climate sensitivity *ab initio*. The canonical range set more than 4 decades ago^74^ is that doubling the amount of CO_2_ should increase the temperature by 1.5–4.5°C. This is also the typical range indicated in the IPCC scientific reports.^75^ However, climate simulations have a very hard time pinning down this number because of the large uncertainties in the feedbacks, especially through changes in the cloud cover.

## RECONSTRUCTING PHANEROZOIC CLIMATE DRIVERS

Once we characterized the main drivers and how they affect the climate, the next step is to reconstruct their variations over the Phanerozoic.

### Reconstructing the atmosphere

The standard yardstick for pCO_2_ temperature reconstruction is the GEOCARB model,^76^ later expanded in the GEOCARB‐SULF model to describe the carbon, sulfur, and oxygen cycles.^77^, ^78^ The model includes several dozen parameters and integrates various proxy measurements of CO_2_. Other greenhouse gases, such as methane or ammonia, are significantly less constrained, either theoretically or through observations. On a multi‐thousand‐year time scale, the atmospheric concentration of CO_2_ and CH_4_ can be directly measured from ice cores, but over the Phanerozoic, the concentration of CO_2_ must be reconstructed from a variety of proxies, each with a large degree of uncertainty.^78^ Nonetheless, there is a very clear correlation between CO_2_ and CH_4_ levels observed in ice cores, such that the total radiative forcing of both gases is about 20% larger than CO_2_ alone. If similar correlations exist over the Phanerozoic time scale as well, then it would introduce biases that might be difficult to unravel. More about this is covered in the discussion below. All things considered, this implies that we are left with a large uncertainty regarding the drivers of atmospheric radiation.

### Solar luminosity increase

The solar luminosity increase is relatively straightforward to reconstruct. It is based on well‐tested solar models satisfying very stringent constraints on the luminosity today, surface abundances, and helioseismology observations. Gough^3^ has shown that the solar luminosity at time *t* before the present can be written to a very good approximation as *L(t)/L = (1 + 0.4t/t*_⊙_), with *t* being the time relative to today (which is negative in the past), and *t*_⊙_ = 4.57 Ga, except for the first 200 Ma of the solar system's life. This was corroborated by Bahcall et al., who found a 5% linear increase over the past 600 Ma.^4^

### Reconstructing CR flux variations

Once meteorites break off their parent body, their surfaces interact with CRs producing spallation products. Some of these products are radioactive, while others are stable. The ratio between the two spallation products provides the integrated CR flux that the meteorite was exposed to between its formation and penetration into Earth's atmosphere. The standard assumption is that the CRF is roughly constant such that the integrated flux corresponds to the exposure age of the meteorite. This assumption, however, leads to an inconsistency between exposure ages derived from short‐lived radionucleotides, such as ^10^Be, and exposure ages based on ^40^K, which has a half‐life of 1.25 Ga. This led to the conclusion that the CRF must vary over geological time scales, being about 30% higher over the past few Ma than its average over the past 1 Ga.^79^

It was then proposed that the exposure age of meteorites can actually be used to reconstruct the CRF history.^57^ If one assumes, statistically, that meteorites are produced at roughly a constant rate, then the distribution of their exposure ages provides an estimate of the time‐varying CRF. It was found that the CRF exhibits a 145 Ma oscillation over the past 1000 Ma. Given that there are very few older iron meteorites, it is impossible to extend the CRF reconstruction further in time using this approach. Also, the limited number of iron meteorites and the relatively few exposure age determinations mean that age estimates finer than a few 10 Ma are not possible. It was shown that the ∼145 Ma periodicity in CRF corresponds to the passage of the solar system through one of the two sets of spiral arms of the Milky Way^58^ (a four‐armed set that extends from our galactocentric radius outward, and which is rotating at roughly half the angular speed that the solar system does).

To reconstruct the variations of CRF on other time scales, it is necessary to resort to theory. On the several 10's of millions of years time scale, we expect CRF variations to arise from the vertical oscillation of the solar system perpendicular to the galactic plane. Observations of the kinematics of A and F stars (which have an intermediate age, sufficient to have reached kinematic equilibrium in the galactic potential perpendicular to the disk, but not too old to be too faint or to have strayed to large vertical distances) give a half crossing period of typically 30–45 Ma.^80^, ^81^, ^82^ The problem, however, is that such kinematic methods suffer from a large systematic bias arising from the spiral arm shocks perturbing the distribution of the stellar velocities during each spiral arm passage.^83^ Thus, we do not have independent CRF reconstructions arising from the vertical motion. Nonetheless, we do have two consistency checks that the paleoclimate temperature reconstruction should satisfy. Apart from a rough range of periods, the phase should be close to peak coldness given our location close to the galactic plane. Moreover, a secondary oscillation arising from the radial epicyclic motion of the solar system, having a period of typically 180 Ma, should manifest itself as an oscillation in the period of the vertical oscillation. Both consistency checks are satisfied by the data.^59^

## COMPARING THE DRIVERS TO THE CLIMATE

Armed with the Phanerozoic temperature reconstructions (Ref. 44; this study, see Figure 1) that minimize the problems of either the lithological or the geochemical data, we can now compare it to the climate drivers. To do so, we use a model that parametrizes the drivers and the temperature reconstructions, while considering the uncertainties. The predicted model temperature assumes greenhouse forcing by CO_2_, heating by the slowly brightening sun, as well as two oscillatory components that describe the effects of spiral arm passages and the vertical motion of the solar system. We also assume that the geochemically measured temperature has a bias through the aforementioned pH alteration of δ^18^O. Thus, we write:

$$\begin{array}{ccc} T_{\mathrm{pred}} & = & T_{0}+\Delta T_{\times 2}\log_{2}\left(\frac{\mathrm{pC}O_{2}}{p_{0}}\right)-\alpha \Delta T_{\times 2}R_{L}t+\frac{A_{l}}{2}\cos\left(\frac{2\pi t}{P_{l}}-\phi_{l}\right)\\ & & +\;\frac{A_{s}}{2}\cos\left(\frac{2\pi }{P_{s}}\left(t+\frac{A_{2\mathrm{nd}}}{2}\cos\left(\frac{2\pi t}{P_{2\mathrm{nd}}}-\phi_{2\mathrm{nd}}\right)\right)-\phi_{s}\right)\\ T_{\mathrm{bias}\mathrm{ed}} & = & T_{\mathrm{pred}}+\Delta T_{\mathrm{bias}}\log_{2}\left(\frac{\mathrm{pC}O_{2,\mathrm{fast}}}{p_{0}}\right). \end{array}$$

*T*_0_ is the temperature for the fiducial parameters of the forcing. *∆T_×_*_2_ is the global temperature increase per CO_2_ doubling. *p*_0_ is the preindustrial atmospheric concentration. *R_L_* is the rate at which the solar radiative forcing increases. *α* is the ratio between the sensitivity to solar forcing and CO_2_. Naively, one would expect this number to be unity; however, various additional effects could enter. A_l_ and A_s_ are the peak‐to‐peak amplitudes of the long‐term and short‐term oscillations (presumably due to spiral arm passages and oscillations perpendicular to the galactic plane). *P_i_* and *ϕ_i_* are oscillation period and phase, respectively. Note that the short‐term oscillation also includes a phase oscillation. *T_biased_* is the biased temperature, and it depends on *∆T_bias_*, which is also a free parameter.

The analysis methodology is as follows:

1. We first generate a combined temperature reconstruction as described in the third section of the second part of this review.
2. For a given set of model parameters, we predict unbiased and CO_2_/pH–biased temporal curves for the temperature.
3. We then find the model parameters that minimize the χ^2^ difference between the model temperature and the reconstructed temperature curve. The minimization procedure uses a combination of simulated annealing to scan the parameter space for minimums to find the global one, and then uses the steepest gradient method to accurately pinpoint the minimum.
4. For the error analysis, we use the bootstrap method. We degrade the data by randomly discarding 1/*e* of the data and repeating the reconstruction and then the minimization procedure. For the high‐resolution geochemical data,^29^ we assume each 1 Ma data point is uncorrelated. For the geochemical/lithological reconstruction, we use 15 Ma bins.
5. The fit procedure is independently carried out to the Scotese geochemical/lithological temperature curve (dashed curve, Figure 2) and to the lithological/geochemical temperature curve of this study (solid curve, Figure 2). The results are summarized in Table 1.

**TABLE 1:** Model parameters fitting the Phanerozoic

Parameter	Meaning	Scotese model	Present model	Significance
**ΔT_×2_**	Sensitivity to CO_2_ doubling	1.52 ± 0.3°C	1.67 ± 0.22°C	7.6 σ
**λ**	Sensitivity to solar flux	0.96 ± 0.09°C/Wm^2^	0.89 ± 0.16°C/Wm^2^	10.2 σ
**α**	Solar warming to CO_2_ ratio	1.95 ± 0.25	1.91 ± 0.12	
**ΔT_bias_**	Indirect CO_2_ bias of δ^18^O	−	−0.07 ± 0.62	
**A_long_**	Large oscillation/amp.	7.2 ± 0.8°C	6.8 ± 0.4°C	16.7 σ
**P_long_**	Large oscillation/period	140.2 ± 1.7 Ma	140.0 ± 0.8 Ma	
**ϕ_long_**	Large oscillation/phase	214 ± 9°	220 ± 5°	
**A_short_**	Short oscillation/amp.	2.6 ± 0.4°C	2.6 ± 0.3°C	7.9 σ
**P_short_**	Short oscillation/period	32.7 ± 0.8 Ma	30.7 ± 0.7 Ma	
**P_short_**	Short oscillation/phase	203 ± 50°	117 ± 40°	
**A_2nd_**	Modulation/amp.	18.4 ± 8°C	21.6 ± 7°C	3.0 σ
**P_2nd_**	Modulation/period	181.5 ± 6 Ma	175 ± 3 Ma	
**ϕ_2nd_**	Modulation/phase	80 ± 48°	66 ± 61°	

*Note*: Errors are 1–σ ranges.

The first outcome of the table is that the fit to the lithological/geochemical temperature curve of this study yields model parameters that are similar to the fit for the Scotese temperature curve, though the results of this study have generally smaller errors. The last column of the table quantifies the significance of the derived model parameters arising from the lithological/geochemical temperature curve of this study. Clearly, then, we find statistically significant signatures to the CO_2_ radiative forcing (at 7.6 σ), as well as to the periodic signals associated with the spiral arm passages (at 16.7 σ) and to the vertical oscillation of the solar system (at almost 7.9 σ).

**FIGURE 3:**
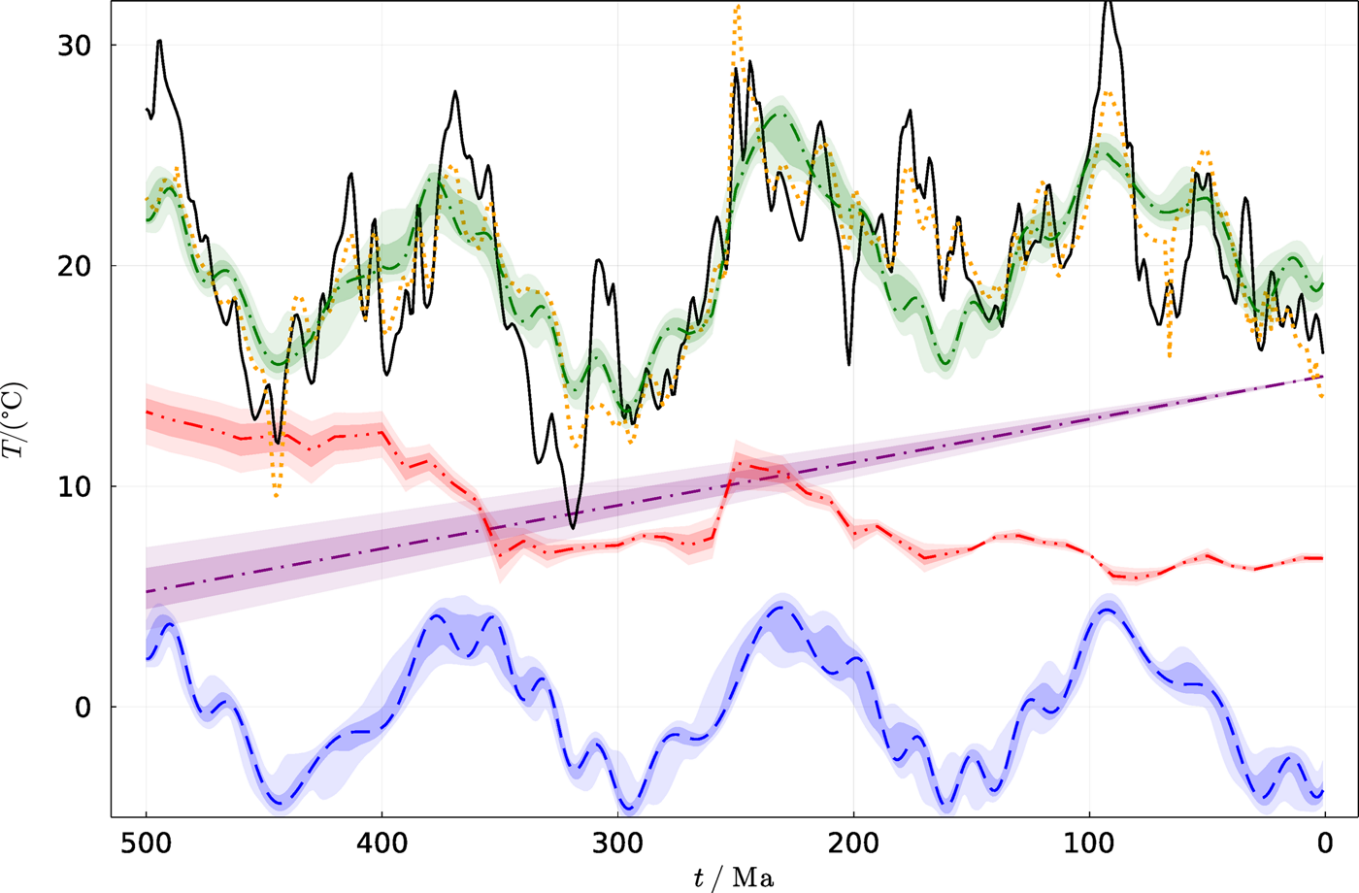
Phanerozoic average global temperature. Plotted are the geochemical/lithological reconstruction of Scotese et al.^44^ (dotted) and combined geochemical/lithological reconstruction (this study, solid), as well as the modeled temperature (dash‐dotted, green). The additional graphs are the different components in the model: atmospheric ionization (bottom, dashed), CO_2_ (dash–double‐dotted), and increasing solar luminosity (dash‐dotted, purple). The shaded regions are 1‐σ and 95% confidence error regions.

**FIGURE 4:**
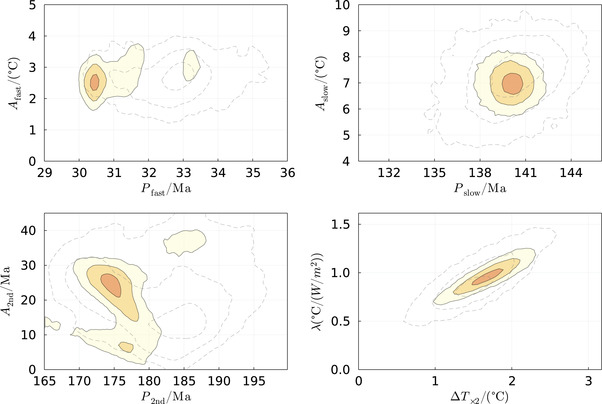
Distribution of model parameter pairs when the rest are marginalized. Dashed and shaded contours are based on the Scotese model and combined data of this study, respectively. Top left: The amplitude and period of the fast oscillation (presumably the vertical motion of the solar system). Top right: The amplitude and period of the slow oscillation (presumably the spiral arm passages). Bottom left: The amplitude and period of the secondary period modulation of the fast oscillation (presumably due to radial epicyclic motion of the solar system in the galaxy). Bottom right: *∆T_×_*_2_ is the climate sensitivity to changes in CO_2_, and $\lambda \equiv \alpha \Delta T_{\times 2}$, which is the sensitivity of the global temperature to the solar luminosity increase (see text).

**FIGURE 5:**
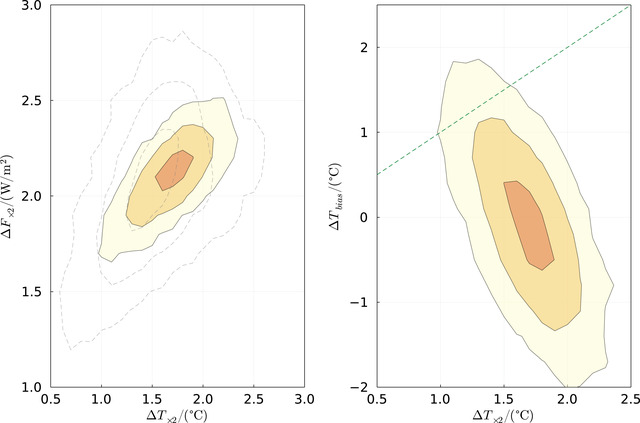
Left: The distribution of the radiative forcing of CO_2_ doubling and climate sensitivity to CO_2_ doubling, mostly constrained by the model fit to both the solar brightening and CO_2_ variations. Right: The likelihood of the climate sensitivity (*∆T_×_*_2_) and the CO_2_ bias parameter (*∆T_bias_*, which quantifies the systematic bias that CO_2_ has on the δ^18^O‐based temperature) when other model parameters are marginalized. The dashed line corresponds to a bias for which the pH and temperature effects on δ^18^O cancel out to give no CO_2_/δ^18^O correlation.

The results are also depicted in Figures 3, 4, 5. Figure 3 depicts the two temperature constructions (Ref. 44 and this study) as well as the model fit (Figure 3, green dash‐dotted line). It is evident from the figure that the three climate drivers used in the model (i.e., CO_2_ radiative forcing [dash–double‐dotted line], atmospheric ionization [dashed line], and solar luminosity [purple dash‐dotted line]) explain most of the temperature variations observed during the Phanerozoic, at least on time scales longer than ∼10 million years.

Figure 4 depicts 2D probability distribution functions for a few parameter pairs when the rest are marginalized. The first three pairs are the amplitude and period of the long and the short oscillations, and the secondary phase modulation of the short oscillation. The dashed lines correspond to the fit to the Scotese model, while the colored contours correspond to the fit to the combined data of this study. The last pair describes the solar forcing sensitivity and the sensitivity to CO_2_ doubling. Because the long‐term decrease in CO_2_ mostly cancels the increasing solar luminosity, they should have a linear relation, giving the narrow oval contours. The finite length of these contours arises from the fact that the CO_2_ is not entirely monotonic such that its contribution can still be fingerprinted.

Because the long‐term decrease in CO_2_ should mostly offset the increase in the solar luminosity, given that both temperature curves do not exhibit any net temperature trend over the entire Phanerozoic, one can relate the CO_2_ forcing in terms of the changing solar luminosity. Since the solar luminosity increase is very well determined from solar evolution models, we can quantify the CO_2_ forcing. This is depicted in the left panel of Figure 5. We find that the CO_2_ should be 2.1 ± 0.1 W/m^2^ per CO_2_ doubling. This should be compared with the 3.7 W/m^2^ per CO_2_ doubling obtained from radiative transfer models,^55^ suggesting perhaps that the CO_2_ has a radiative forcing that is smaller than expected, or that there are unaccounted systematic errors.

The right panel of Figure 5 depicts the quantification of the possible bias in the geochemical data, which arises from the CO_2_ → pH → δ^18^O link. We find no appreciable bias, which suggests that the oceans have had a relatively strong buffering effect on the pH variable.

## DISCUSSION AND SUMMARY

In the second part of this review, we discussed a novel type of temperature record that combines lithological evidence of climate with geochemical (δ^18^O) temperature measurements. There are distinct advantages to this combined approach because the data sets are completely independent of each other and, therefore, the errors are orthogonal. The temporal resolution of lithologically based paleo‐Köppen belts is poor; however, these reconstructions are immune to certain systematic biases that exist in the geochemical records. The first is the long‐term drift in the baseline δ^18^O–temperature calibration. The second is the fact that δ^18^O can, in principle, depend on the atmospheric CO_2_ levels (through the effects of ocean acidity), implying that pCO_2_ → ∆T →δ^18^O may not be the only route affecting the isotopic record. By combining the best attributes of both methodologies, it is possible to produce a hybrid data set that can overcome these problems. Guided by the long‐term lithological record, it relies on the δ^18^O record to provide short‐term variations.^44^

We also reviewed the major global climate drivers that appear to operate over the Phanerozoic. These include drivers that are intrinsic to the Earth's system and drivers that are extrinsic to it. We expect the former group to include several drivers, such as greenhouse gases, changing continental distribution, albedo variations, and more. However, the only driver we can reliably estimate when modeling Phanerozoic temperature is CO_2_. As for the other drivers, some may be important, but they are either difficult to reconstruct (such as other greenhouse gases), or their global effect is difficult to assess quantitatively (such as changing continental geography). In the best‐case scenario, some of the temperature variations are left unexplained. The more problematic scenario involves drivers that may correlate with CO_2_. For example, CH_4_ correlates with CO_2_ in the ice core–based records over the past several hundred thousand years; as a result, the overall CO_2_ forcing is effectively increased by about 20%. Such a correlation over the Phanerozoic would give rise to systematic errors that would affect the conclusions described below.

Besides the intrinsic factors, we also expect extrinsic factors to influence Earth's climate as well. On “short” time scales, it is the Milankovitch cycles, which, however, are too rapid to be seen on the time scales employed in this study (i.e., millions of years). However, we expect the slowly changing galactic geography to have had an effect on the Earth's climate through modulation of ionization in the Earth's atmosphere. Today, we know from multiple approaches, ranging from empirical evidence to theory with supporting measurements in the lab, that atmospheric ionization governs the formation of small condensation nuclei. A higher CR flux would produce a greater degree of atmospheric ionization and would result in the formation of more small condensation nuclei. This, in turn, increases the probability that those small nuclei will become CCNs, producing whiter, longer‐living clouds.

The largest variations in CR flux arise from the periodic passage of the solar system through the galactic arms. This is reflected in the exposure ages of meteorites, which exhibit a roughly 145 Ma periodicity over the past billion years. And indeed, every one of these high CRF epochs corresponds to a lithologically documented ice‐age episode, including the Neoproterozoic Snowball Earth. On shorter time scales, one expects a temperature oscillation to arise because of the solar system's motion perpendicular to the galactic plane. Although the CR flux cannot be directly reconstructed on this shorter time scale, we do see a 32 Ma oscillation in temperature during the past 500 Ma. In the climate model presented here, we propose that these oscillations are possible climate drivers.

The last important driver that we consider is the slow, steady increase in solar luminosity with time. This complicates the analysis because it compensates for some of the CO_2_ radiative forcing that has significantly decreased over the same interval. Namely, we have to rely on the nonmonotonic variations of the CO_2_, if we are to find the fingerprint of either the CO_2_ or the decreasing solar luminosity in the temperature model.

Armed with the above drivers, we have seen that one can build a model that explains a significant part of the temperature variations observed over the Phanerozoic. This model has unexplained residuals of only a few °C. Each of the three principal drivers (CO_2_ variations, atmospheric ionization changes arising from passages through the galactic arms, and the secular increasing of the solar luminosity) provide comparable contributions of about 7–8°C to the temperature model. Interestingly, however, decreases in CO_2_ concentration and the increase in solar luminosity mostly cancel each other out (except for the nonsecular variations in CO_2_). Consequently, the dominant temperature variations observed during the Phanerozoic are those due to the periodic passages of the solar system through the galactic spiral arms. The fourth important component is the vertical motion of the solar system perpendicular to the galactic plane, which is about one‐third of the other contributions. These nonmonotonic radiative forcings (both intrinsic and extrinsic) have relative error bars of ∼10% or less, implying that their presence in the data is confirmed to have a high statistical significance. However, some caveats should be mentioned.

Although the CO_2_ forcing is detected with very high significance, there are systematic biases which we have not considered. The CO_2_ levels taken were the nominal values of the GEOCARB‐SULF model.^77^ If CO_2_ levels are systematically lower/higher, then the inferred climate response to CO_2_ levels should be correspondingly higher/lower than the ∆*T*_×2_ ≈ 1.7 ± 0.25°C per CO_2_ doubling derived here. A second bias, mentioned above, can arise if there is an additional greenhouse gas forcing by a gas that correlates with CO_2_ levels. In such a case, the climate sensitivity would be correspondingly lower. For example, the 20% increase in forcing resulting from a CH_4_/CO_2_ correlation, similar to that seen in ice cores, would decrease estimates of climate sensitivity to 1.35 ± 0.25°C per CO_2_ doubling. We also note that this climate sensitivity includes all responses, including long‐term ones, such as the albedo variations associated with long‐term changes in the glaciations, which are generally not considered as part of the climate sensitivity on the centennial time scale.

Another interesting conclusion is that the δ^18^O could, in principle, be used to measure the CO_2_ radiative forcing. This is because its long‐term decrease should mostly compensate for the secular solar luminosity increase to preempt any long unidirectional temperature trend over the Phanerozoic. This gives a radiative forcing which is ∼2/3 of the canonical value obtained from line‐by‐line radiative transfer models. Thus, although we covered in this review the largest contributions to climate variations over the Phanerozoic, each one with a statistically significant fingerprint, several questions still remain open.

## AUTHOR CONTRIBUTIONS

Most of the geology aspects were contributed by J.V., while most of the astrophysics was contributed by N.J.S. The analysis was done primarily by N.J.S., with input from J.V and H.S. The manuscript was written by N.J.S., H.S., and J.V.

## COMPETING INTERESTS

The authors declare no competing interests.

### PEER REVIEW

The peer review history for this article is available at: https://publons.com/publon/10.1111/nyas.14920.

## Supporting information

Supporting
